# Global waterfowl production: stocking rate is a key factor for improving productivity and well-being—a review

**DOI:** 10.1007/s11250-023-03835-6

**Published:** 2023-11-24

**Authors:** Mohamed I. El Sabry, Obaida Almasri

**Affiliations:** Department of Animal Production, Faculty of Agriculture, Cairo University, 6 El-Gamma St, Giza, 12613 Egypt

**Keywords:** Duck population, Space allowance, Growth rate, Egg production, Meat production, Carcass weight, Meat quality

## Abstract

Waterfowl is an important animal-protein source, which has the potential to get a bigger share in the animal production sector. However, waterfowl farming practices and welfare standards are not well established yet. Stocking rate is one of the farming standards that can enhance the productivity, behavior, and well-being of birds; however, rare studies are available in this area. Thus, this article (1) gives an overview of the recent global waterfowls’ meat and egg production and their population distribution, (2) reviews the effects of stocking rate on social, feeding, and sexual behaviors, (3) shows the effects of stocking rate on growth performance, carcass weight, and meat quality of ducks and geese, and (4) declares the relationship between the stocking rate and egg production. Conclusively, an optimal stocking rate standard can improve behaviors, productivity (meat-egg), and meat quality. Moreover, using weight (kg)/m2 will help in affording the required space allowance for different ducks and geese under various housing systems. The fish-waterfowl production system could be a promising and sustainable solution for increasing waterfowl production, maintaining the welfare of birds, saving energy, and reducing the water footprint of waterfowl meat. Based on prior research findings, we recommended adopting the stocking rate (SR) standard for specific duck and goose breeds to achieve an optimal production-welfare balance.

## Introduction

Domestic ducks trace back to two species of the mallard duck (*Anas platyrhynchos*) and the Muscovy duck (*Cairina moschata*), while the origins of geese are the greylag goose (*Anser anser*) and the swan goose (*Anser cygnoides*). Housing waterfowl has some advantages such as (1) low feeding cost and feeding on cheap byproducts in rural areas. Consequently, utilizing such feed sources reduces the competition between waterfowl and human nutritional resources. (2) Keeping waterfowl in fish ponds could increase their economic efficacy by reducing feeding costs by increasing the amount of plankton as feed for fish (Jiang et al. (2011). Also, this kind of housing method may reduce the water footprint of waterfowl products, which is becoming a critical challenge in water-stressed countries. (3) Adaptability to tropical and subtropical climates, which are hot and humid (Farghly et al. 2019). For example, European goose breeds, such as the Embden and Toulouse, have been introduced into tropical countries with notable success (Pingel 2009). (4) Higher resistance to disease compared to other poultry species. (5) Suitability for integrated farming systems since waterfowl can be used for controlling weeds as relish grasses and most broad-leaved plants in many crops, where pesticides cannot be used (Patil et al. 2021). Compared to the characteristics of other poultry species, Pingel (2011) suggested that the increase in waterfowl production could reduce the supply-demand gap in animal protein and improve food security, especially for rural families. In this context, the duck population has increased; e.g., in 2010, the duck population was 2.1 billion ducks, and 4 million tonnes of duck meat were produced (FAO 2010). Yahoo Finance (2022) referred to the increase in waterfowl production by 3% yearly from 2010 to 2021. At the same time, goose farming has become economically important in Asia and central Europe (Eda et al. 2022 )

On the other hand, the majority of the housing system investigations focused on the behavior, well-being, and productivity of chickens rather than ducks and geese. Furthermore, management recommendations for growing ducks and geese were inconsistent due to the interaction of several experimental factors such as the genetic background of the breed (Abdel-Rahman et al. 2008), size of groups, different housing systems (Caroprese et al. 2009), and water quality (El Sabry et al. 2023a). So, standards of housing and welfare for waterfowl require more improvement, which will help in enhancing the revenue of duck and goose farming.

In this regard, the welfare-cost balance becomes one of the animal production aspects; the stocking rate of livestock and poultry should be considered for improving the productivity, health, and well-being of animals and birds (El Sabry and Almasri 2022; European Food Safety Authority 2023). Moreover, the species-specific behavior can be altered due to the stocking rate (SR), housing system, and environmental conditions (El Sabry and Almasri 2023; El Sabry et al. 2023b). Many studies reported that the high SR adversely affects the productivity and reproductivity of poultry, which is due to numerous suggestions: (1) decreased area for each bird, consequently forcing the birds to stand and increased their energy requirements and decreased the ability to rest, (2) high-temperature stress of the birds per unit, (3) inadequate air exchange, (4) high ammonia, (5) moreover, high SR deteriorated the duck’s antioxidant capacity, consequently interrupt the antioxidant defense system and finally caused oxidative stress. Moreover, high SR increases the heat increment, which negatively affects ducks’ performance, even if birds have enough on the feeders (Li et al. 2017; Bawish et al. 2018a; Nasr et al. 2022).

Therefore, this review sheds light on (1) waterfowl meat and egg production, (2) the geographical distribution of duck and goose population, (3) the effects of SR on the social, feeding, and feather pecking behaviors, (4) the effects of SR on growth performance, carcass weight, and meat quality of ducks and geese, and (5) the relationship between stocking rate and egg production.

## Geographical distribution of the duck and goose populations

**Fig. 1:**
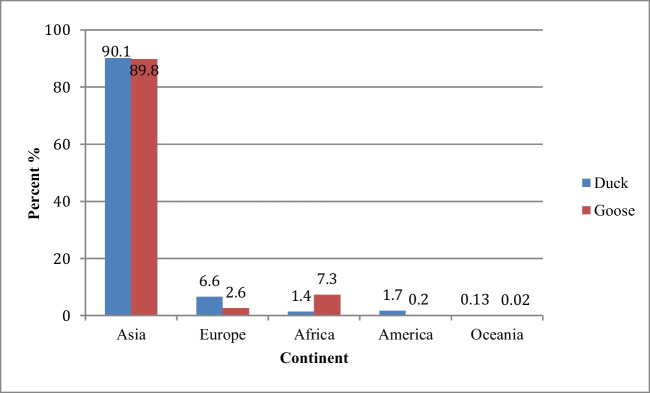
The distribution of duck and goose population in different continents

**Fig. 2:**
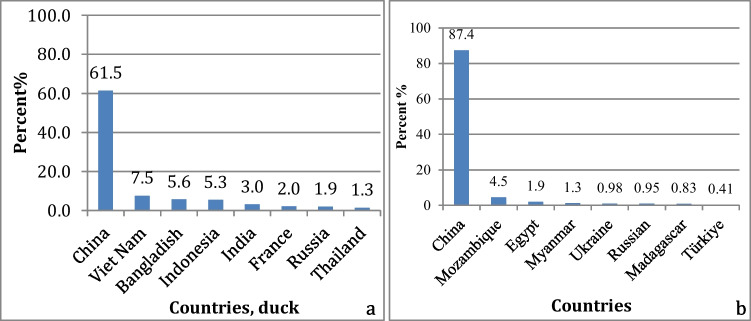
The distribution of (**a**) duck and (**b**) goose population in different countries

The total size of the duck populations all over the world is about 1,099,332,000 head, and most of them are distributed throughout Eurasia. The largest duck populations are found in Asian countries (see Figs. 1 and 2). Regarding the goose population, there were 363,132,000 geese worldwide about 89.8% of the goose population is found in Asia, 7.3% in Africa, and 2.6% in Europe (Fig. 1). The largest goose population is found in China, followed by other countries in different continents (Fig. 2b) (FAO, 2021).

**Fig. 3:**
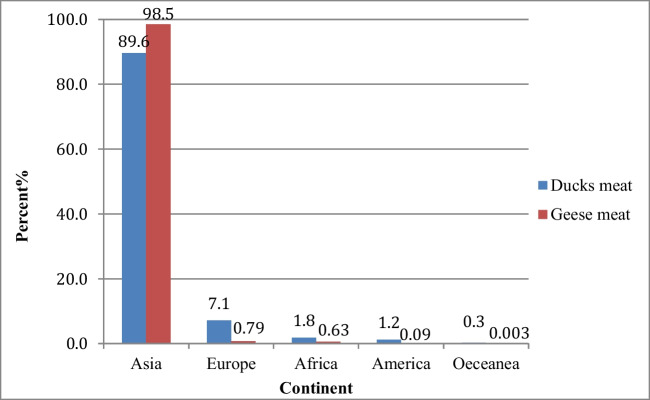
The distribution of duck and goose meat production in different continents

The contribution of duck meat to global poultry meat production is about 4.1%, which is significantly lower compared to the contribution of chicken (Patil et al, 2021), while the statistics of global goose meat production is estimated to be 2.9% compared to chicken meat production. The biggest goose meat production is found in China (98%), followed by Ukraine (0.71%), Egypt (0.33%), and Madagascar (0.29%) (FAO, 2021). The global production of waterfowl meat is summarized in Fig. 3 and Table 1.

**Table 1:** Updated statistics of global duck meat and egg production (million tons)

Rank	Country	Meat production	%	Egg production	%
1	China	4,902,392	79	3,821	83.2
2	Viet Nam	182,651	2.95	70	1.5
3	Bangladesh	60,461	0.97	76	1.7
4	Indonesia	44,198	0.71	208	4.5
5	India	44,000	0.71	Unknown	Unknown
6	France	177,000	2.85	Unknown	Unknown
7	Russia	Unknown	Unknown	Unknown	Unknown
8	Thailand	59,803	0.96	310	6.8
Total	Global production	6,201,639	100	4590	100

Duck eggs are mostly processed into salted eggs, thousand-year eggs (pidan), and balut as the gourmet heritage in several Asian countries. However, the production of duck eggs is still lower than chickens. The consumption of duck eggs accounts for around 10–30% of total egg consumption in East and Southeast Asia (Ismoyowati and Sumarmono 2019). The global egg production of ducks reached 4,590,000 tons in 2009. More statistics about duck egg production are summarized in (Table 1), while there is no available data about the egg production of geese.

According to statistics of FAO (2021), Pingel (2009, 2011), Ismoyowati and Sumarmono (2019), and Patil et al. (2021)

## Impact of stocking rate, group size, and housing system on behavior and welfare indices

Waterfowl can cope with harsh environmental conditions, which results in a misconception that waterfowl do not need a welfare assessment (Xie et al. 2014). Improving the welfare status of waterfowl can be a sustainable strategy for enhancing their performance and health. Space allowance is an environmental factor that directly and indirectly impacts the animal’s behavior, especially under intensive production systems. El Sabry and Almasri (2022; 2023) and El Sabry et al. (2022) mentioned that determining the required SR for farm animals with welfare in mind increases economic revenue.

### Ducks' specific behavior and welfare indices

The housing system and SR can affect the welfare status of farm animals (El Sabry and Almasri 2023; El Sabry et al. 2023b). For, example, using a floor system, from 1 to 70 days old of Muscovy, Bawish et al. (2018a) found that the drinking, feeding, sleeping and resting time, and relaxing behavior (preening %, leg stretching %, tail wagging %, and head shaking %) were significantly better when the SR was reduced from 8 to 4 birds/m^2^. While the feather pecking increased in the ducks that kept in the high SR (8 birds/m^2^). Similar findings by Downing (2022) found the highest probability of feather pecking and skin damage in Cherry Valley and Grimaud Freres Pekin ducks when the SR was increased from 4.4 to 5.2 birds/m^2^.

Using plastic wire-floor pens, Xie et al. (2014) evaluated the effect of SR on the eating and drinking behavior of White Pekin ducks in China. They kept the ducklings at SR of 13, 15, 17, 19, and 21 birds/m^2^ or 8.4, 9.7, 10.9, 11.9, and 13.0 kg of actually achieved body weight (BW)/m^2^ from 1 to 14 days and at SR of 5, 6, 7, 8, and 9 birds/m^2^ or 17.0, 20.3, 23.6, 26.9, and 29.9 kg of actually achieved BW/m^2^ from 14 to 42 days. The result indicated that the high SR negatively affected the eating and drinking behaviors, which may be associated with the decreased feeding and drinking space at high densities.

Regarding locomotor activities, Li et al. (2018) indicated that the flock activity levels and percentage of individuals walking normally of Pekin ducks were significantly affected by different SRs (5, 6, 7, 8, and 9 ducks/m^2^ or 16.3, 19.3, 22.5, 25.5, and 28.5 kg, respectively). They also reported that the highest flock activity and percentage of normally walking individuals were found in the SR (5 birds/m^2^), while it was worse with the increase in SR.

### Geese’s specific behavior and welfare indices

Using a floor system, Kaszynsko et al. (1986) studied the effect of different SRs (2, 3, and 4 birds/m^2^ from 1 day to slaughter age) on feather pecking of the White Italian geese. The results indicated that the lowest feather pecking and highest feather yield were observed from geese at the lowest SR of 2 birds/m^2^.

In plastic wire-floor pens, during the early age (1–14 days), Liu et al. (2021) found that SR of 20 birds/m^2^ is the best for maintaining the feather quality and reducing feather pecking. With the advance in age, Yin et al. (2017a) reported that welfare indices decline with the increase of SR; e.g., when SR was 4 or more birds/m^2^, standing on one leg (relaxing) behavior significantly reduced; when SR was 5 or more birds/m^2^, feather contamination degree and preening behavior both significantly increased; when SR was 6 birds/m^2^, the behaviors of lying and feather pecking, back-feather damage rate, and walking gait problems increased. In the same line, Wang et al. (2019) investigated the effect of different SRs (2.5, 3.5, 4.5, 5.5, 6.5, and 7.5 birds/m^2^) on feather clean of female Sichuan white geese from 49 to 70 days of age. They noted that high SR significantly decreased the feather quality of the back, thoracic-abdominal, wing, and tail, with the advance in age.

### Sexual behavior in ducks

In this context, regarding the effect of different SRs on the welfare and sexual behaviors of ducks, Bilsing et al. (1992) reported that young Muscovy ducks with intact bills on perforated floors showed no feather pecking and cannibalism up to 28 days of age when kept at a lower SR (6.3 birds/m^2^), whereas at a higher SR (11.6 birds/m^2^), feather pecking and injuries occurred. In adult ducks, behavioral disturbances resulted in attacking the females’ cloaca during copulation. Afterward, females not involved in mating attacked the penis of the males (Bilsing et al. 1992). In Table 2, duck and goose-specific behaviors that are influenced by the interaction between space allowance and housing system are summarized.

**Table 2:** The optimal stocking rate in different housing systems for maintaining species-specific behaviors of broiler ducks and geese

Behavior	Strain	Age (wk)	Housing	SR (bird/m^2^)	Country	References
Duck						
Feeding behavior	Muscovy	0–10	Floor litter	4	Egypt	Bawish et al. (2018a)
	White Pekin	0–6	Plastic wire-floor pens	5	China	Xie et al. (2014)
Feather quality	*Cherry Valley *Grimaud Frere Pekin	2–6	Floor litter	4.4	Australia	Downing (2022)
	Muscovy	0–4	Plastic wire-floor pens	6.3	Germany	Bilsing et al.(1992)
Comfort behavior	Muscovy	0–10	Floor litter system	4	Egypt	Bawish et al. (2018a)
Geese						
Feather quality	White Italian	0–10	Floor litter system	2	Italy	Kaszynsko et al. (1986)
	Male Yangzhou	4–10	Plastic wire-floor pens	< 5	China	Yin et al. (2017a)
	Sichuan white	0–2		20		Liu et al. (2021)
		7–10		2.5		Wang et al. (2019)
Comfort walking ability	Male Yangzhou	4–10		4		Yin et al. (2017a)

## Impact of SR on the growth performance traits

### Broiler duck performance

Using the floor-housing system, Taboosha (2014) revealed that at 10 and 12 weeks of age, the BW of female mule ducks was significantly reduced by 8% on average as SR rose to 7 birds/m^2^. Birds raised at SR (5 birds/m^2^) had significantly higher weight gain (WG) than the group of 7 birds/m^2^ during the period from 0 to 12 weeks of age. The feed conversion rate (FCR) value for all groups ranged between 4.2 and 4.5 kg feed/kg live BW. Furthermore, the mortality % was not affected by density. It seems that there is mild distress that hinders WG. In this context, stress-related hormones should be studied to better understand the effect of SR on WG.

Also, in Pekin and Muscovy ducks (10 weeks of age), Ahaotu and Agbasu (2015) and Bawish et al. (2018b) reported that, compared to 8 birds/m^2^, SR of 4 birds/m^2^ increased WG, final BW, decreased feed intake (FI), and improved FCR and dressing percentage, but had no effects on giblets % and mortality %.

In 8 to 41–day-old Cherry Valley and Grimaud Freres ducks, Downing (2022) kept ducks at low, medium, and high SR (4.4, 5.2, and 6 birds/m^2^, respectively) and reported that SR had no effects on final BW, water intake, FI, and FCR. Regarding welfare indices, they concluded that the upper limit for best performance could potentially lie between (5.2 and 6 birds/m^2^ or 16.5–19.0 kg/m^2^).

Using two different litters, wood shaving litter (WSL) or plastic slatted floor (PLS), Abo Ghanima et al. (2020) showed that during the brooding period (1–14 days of age), increasing SR from 9, 15 to 21 birds/m^2^ reduced BW, WG, and FCR, while the FI was increased in both floor types. From the results, it can be seen SR of 9 and 15 are suitable in the case of using WSL, whereas only an SR of 9 is suitable with PLS floors. While at 49 days old, ducklings on the PLS type verified better BW, WG, and FCR compared to the WSL type. Moreover, increasing SR in both floor types significantly reduced the breast, thigh, and left fillet %. They concluded that housing Pekin ducklings on PLS improved their growth and carcass traits, where the optimal SR are 3 and 5 birds/m^2^ using PLS and WSL, respectively. It could be suggested that the possible reason for the superiority of the PLS group could be due to less contact with feces, which maintains better hygiene, and improved air quality due to less litter fermentation.

In plastic wire-floor pens, Xie et al. (2014) studied the effects of SR on growth performance, carcass yield, and foot pad lesions of White Pekin ducks from hatch to 14 days of age (experiment 1) and from 14 to 42 days of age (experiment 2), respectively. In experiment 1, 1-day-old ducks were stocked at SR of 13, 15, 17, 19, and 21 birds/m^2^ or 8.4, 9.7, 10.9, 11.9, and 13.0 kg BW/m^2^, respectively). In experiment 2, ducks that were 14-day-old were stocked according to the SR of 5, 6, 7, 8, and 9 birds/m^2^ or 17.0, 20.3, 23.6, 26.9, and 29.9 kg of actually achieved BW/m^2^, respectively. The results indicated that the final BW and WG of ducks were significantly reduced as SR increased from 19 to 21 birds/m^2^. In addition, the final BW and WG of growing ducks significantly decreased when SR was 9 birds/m^2^. On the other hand, increasing SR did not markedly influence the carcass, breast meat, leg meat, abdominal fat, and foot pad lesions of growing ducks (*P* > 0.05). The authors concluded that the maximum SR from hatch to 14 days of age and from 14 to 42 days of age should not exceed 19 and 8 birds/m^2^ or 11.9 and 26.9 kg of achieved BW/m^2^, respectively. Whereas, the FCR and mortality % were not affected by SR.

In Pekin ducks, Zhang et al. (2018) found that during the period from 22 to 42 days of age, SR should be between 5 and 8 birds/m^2^ or 13.5 and 20.6 kg/m^2^. However, the final BW and average daily gain (ADG) were significantly higher in the lower SR group, while the FCR and meat quality of the groups were similar.

While Li et al. (2018) concluded that the SR should be less or equal to 7 birds/m^2^ or 22.5 kg BW/m^2^.

Also, in Korean native duck breed, from 1 to 50 days, Hong et al. (2019) indicated that the BW and average daily BWG (g/bird/day) were not significantly affected by density (4, 5, 6, 7, 8, and 9 birds/m^2^). But the uniformity %, average daily FI, and FCR impaired significantly as the SR increased. They concluded that the best SR was approximately 7 birds/m^2^ regarding the productivity and better uniformity % of the flock.

In Pekin ducks (1–42 days), Eratalar and Okur (2018) and Eratalar et al. (2022) showed that increasing SR (3, 5, and 7 birds/m^2^) significantly reduced the BW, WG, FI, FCR, productivity index, and carcass weight. Also, the highest slaughter weight was observed in the low SR group compared to the medium and high SR groups. Whereas, mortality % and meat quality parameters, i.e., pH, water-holding capacity, dry matter, and cooking loss, were not affected by different SRs.

In 1 to 70–day-old Muscovy and Mallard ducks, Nasr et al. (2022) revealed that the BW, ADG, carcass weight, and dressing % of both duck breeds were reduced by increasing SR (5, 7, and 9 ducks/m^2^). The authors concluded that the best SR for Muscovy and Mallard ducks is 5 birds/m^2^.

### Goose performance

Using a wire-floor pen, Liu et al. (2021) studied the effect of different SRs (15, 20, 25, 30, and 35 birds/m^2^) on the growth performance of 1 to 14–day-old Sichuan White geese. The results showed that ADG and ADFI were significantly reduced as SR increased from 15 to 35 birds/m^2^, but FCR was not affected at early age. The authors recommended that the SR of geese from 1 to 14 days of age should not be more than 20 birds/m^2^. The authors attributed that the birds raised at a high SR have more difficulty accessing feeders and drinkers.

Also, in female White Sichuan geese, Xue et al. (2021) kept the geese at SR of 4.76, 6.00, 7.33, 8.67, 10.00, and 11.33 birds/m^2^ from 14 to 28 days and at SR of 2.50, 3.75, 5.00, 6.25, 7.50, and 8.75 birds/m^2^ from 28 to 49 days. Results of the first experiment indicated that the increase of SR, BW, and WG significantly decreased in the experiment. It also significantly impaired FI and FCR. In the second experiment, as the SR increased, the BW and WG decreased (*P* < 0.05), while FCR increased linearly (*P* < 0.05). They concluded that the upper critical SR of geese from 14 to 49 days of age for WG and FCR was (4.76–4.8 birds/m^2^). However, the results of FI, WG, BW, and FCR during the period from 28 to 49 days of age were not affected at SR of 6.25 birds/m^2^. There is a kind of contradiction in the results of the two experiments; thus, we assume that this SR of 6.25 birds/m^2^ is also acceptable.

Lin et al. (2016) investigated the effect of SR on the growth traits of White Roman geese in Taiwan. They stocked the ducklings at SR of 24, 32, and 40 birds/1.92 m^2^ or 12.5, 16.7, and 20.8 kg BW/m^2^ from 1 to 3 weeks; at SR of 24, 32, and 40 birds/13.2 m^2^ or 4.64, 6.18, and 7.73 kg BW/m^2^ from 4 to 6 weeks; and at SR of 24, 30 and 36 birds/20 m^2^ or 5.75, 7.18, and 8.62 kg BW/m^2^ from 7 to 14 weeks. The results indicated that the SR mainly affected BWG in geese younger than 4 weeks and that geese stocked at low SR had a high BW at 2 weeks of age. At the 14 weeks, the results revealed that the BW, FI, and WG significantly reduced with an increased SR, while the FCR was not affected.

In 28-day-old, male Yangzhou goslings, Yin et al. (2017b) showed that with the increase of SR from 2 to 6 birds/m^2^, the BW of geese at 42 and 70 days were significantly reduced by about 10.4%. Whereas the FCR during the period from 28 to 70 days of age significantly increased from 3.80 to 4.24. However, ADFI and mortality % were not affected by the different SRs. The authors recommended that the SR of geese should be kept to 5 or fewer birds/m^2^.

In female Sichuan white geese, Wang et al. (2019) showed that the ADG was significantly reduced with an increase of SR (2.5, 3.5, 4.5, 5.5, 6.5, and 7.5 birds/m^2^), While, the FCR impaired with an increase of SR during the experimental period (49 to 70 days). Whereas, the SR did not influence slaughter yield %, breast meat yield %, leg meat, subcutaneous fat and skin, and abdominal fat at 70 days of age. The authors concluded that the optimal SR of female Sichuan white geese is 3.5 birds/m^2^ or less. Also, in males of Sanhua geese, Ying et al. (2021) found that as the SR increased from 2 to 5 birds/m^2^, the BW of geese at marketing age (70 days) was significantly reduced by 15.9%. The higher FI and better FCR were in the low SR group compared to those in the high SR group. Whereas, no effect on the mortality % was recorded.

Using a floor system, Kaszynsko et al. (1986) kept the goslings at SR of 7, 10, and 13 birds/m^2^ from 1 to 21 days; at SR of 3, 4, and 5 birds/from 22 to 56 days; and at SR of 2, 3 and 4 birds/m^2^ from 57 days to slaughter age. The results indicated that the WG, FCR, and carcass quality of the White Italian geese in high SR (4 geese/m^2^) were lower compared to those in low SR groups (2 and 3 geese/m^2^).

## Effect of stocking rate on egg production of waterfowl

The egg production traits are affected by genetics, nutrition, diseases, heat stress, water restriction, and SR. Mehaisen et al. (2016), Abbas et al. (2022), and El Sabry et al. (2023b) reported that these factors directly or indirectly affect physiological functions and immune system development. Interesting findings demonstrated that high SR reduces sex hormone levels. Consequently, low level of sex hormones retards ovarian development and causes follicular atresia that lead to poor laying performance (Cai et al. 2019). However, no standard SR has not established yet for the ducks and geese.

### Egg production of ducks under different SRs

In this regard, under a housing system (semi-openly on land and water), Jiang et al. (2022) investigated the effects of various SR (5, 10, and 15 birds/m^2^) on the development and maturation of ovary, and the mRNA expression of key genes in the reproductive axis during the rearing period in 180 healthy 7-week-old Shan-ma ducks. The results indicated that the gonad index and degree of ovarian development of the egg-laying ducks significantly lessened with increasing SR, and the ovarian development and maturity were retarded in the medium and high SR. Therefore, rates of ovarian development and maturation could be reduced by a high SR leading to a delay in reproductive performance during the rearing period of Shan-ma ducks.

In China, Xiong et al. (2020) studied the effects of different SRs of 4, 5, 6, 7, and 8 birds/m^2^ on egg quality and antioxidant capacity of Jinding laying ducks (20-week-old) that were kept on plastic wire-floor pens. They reported that a linear increase in the SR reduced egg production %, egg mass, and eggshell strength. The highest egg production %, egg mass, and eggshell strength (88.1%, 57.7 g/day, and 53.4 respectively) were in birds that stocked at low SR (4 birds/m^2^), while the lowest egg production %, egg mass, and eggshell strength (67.1%, 49.9 g/day, and 48.8, respectively) were in birds stocked at high SR (8 birds/m2). Also, the high SR negatively affects FCR. The FCR was (2.8) in birds that stocked at low SR (4 birds/m^2^) compared to (3.4) in birds that stocked at high SR (8 birds/m^2^). Whereas, the egg weight was not affected by different SRs. This contradiction in the egg mass and weight was not justified by the researchers. Therefore, we suggest that 4 laying ducks/m^2^ could be recommended to maintain high egg production % and better egg quality.

In the following, the productive performance of ducks that is influenced by the interaction between space allowance and the housing system is summarized in Table 3.

**Table 3:** The best stocking rate according to the housing system, age (week), and breed of ducks

Breed/strain	Age	Housing	SR (b/m^2^)	Final BW (g)	References
Broiler ducks					
Female of mule duck	0–12	Floor system	5–6	3570	Taboosha (2014)
Muscovy	0–10		4	3348	Bawish et al. 2018a, b)
Cherry Valley and Grimaud Freres Pekin	2–6		5.2–6	3170	Downing(2022)
French Pekin	0–2	Wood shaving litter	9–15	605	Abo Ghanima et al. (2020)
	2–7		3	3385	
	0–2	Plastic slatted floor	9	604	
	2–7		5	3401	
White Pekin	0–2		19	627	Xie et al. (2014)
White Pekin	2–6		8	3362	
Pekin	3–6		5 8	2576 2711	Zhang et al. (2018)
White Pekin	3–6		7	3210	Li et al. (2018)
Korean native	0–7		7	2586	Hong et al. (2019)
Pekin	0–6		3	3263	Eratalar and Okur (2018)
Muscovy Mallard	0–10		5	3882 4114	Nasr et al. (2022)

## Egg production of geese under different SRs

In the “geese-fish” production system, Jiang et al. (2011) conducted two experiments to test the effect of different SRs (0.5 and 0.67 goose/m^2^) on the health and reproductive performance of Magang geese. Results showed that raising SR elevated bacteria counts, lipopolysaccharide (LPS) concentrations in water, and goose plasma and decreased egg fertility and embryonic livability. In addition, it is worth noting that counts of total bacteria, *Escherichia coli* and *Salmonella* in water, as well as water and goose plasma LPS concentrations, tended to decline from the highest levels in summer, to intermediate levels in autumn, and to the lowest one in winter. This was reflected in the trend of embryonic mortality and hatchability %. Conclusively, maintaining high water hygiene, goose productivity, and gosling quality could be achieved by providing 2 m^2^ of water surface for each goose.

In the following, the productive performance of geese that is influenced by the interaction between space allowance and the housing system is summarized in Table 4.

**Table 4:** The optimal stocking rate for goose strains at different ages (week) under different housing systems

Breed/strain	Age	Housing	SR (b/m^2^)	Final BW (g)	References
Sichuan White	0–2	Plastic wire-floor pens	20	-	Liu et al. (2021)
	2–4		4.76	1355	Xue et al. (2021)
	4–7		4.83		
	7–10		3.5		Wang et al. (2019)
White Roman	0–14		≤ 5		Lin et al. (2016)
Yangzhou	4–6		3	2331	Yin et al.(2017b)
	6–10		2	3923	
White Italian	8–10		2–3		Kaszynsko et al. (1986)

Finally, according to the available studies, we suggested the standard SR for some breeds of ducks in Table 5.

**Table 5:** Suggested SR standards for maintaining a production-welfare balance of ducks under intensive production systems

Breed/strain	Age (wk)	SR (bird/m^2^)	Total BW/m^2^ at marketing age
Pekin	0–2	15	9.1
	2–7	5	17
Muscovy	0–10	5	18.1
Mallard	0–10	5	20.6
Korean	0–7	7	18.1
Cherry Valley	0–7	5	15.8
Mule	0–12	5–6	17.9–21.4
Jinding	20–40	4	-

Regarding the standard SR for some breeds of geese, we suggested for Sichuan White geese from 0 to 2 weeks, from 2 to 4 weeks, and from 7 to 10 weeks, the standard SR is 20, 4.8, and 3/m^2^, respectively.

Considering an economic-welfare balance for some breeds of geese, the suggested standard SR ranges are as follows: for Sichuan White geese, from 0 to 2, 2 to 4, and 7 to 10 weeks, the standard SR are 20, 4.8, and 3/m^2^, respectively. For White Roman geese, from 0 to 14 weeks, the recommended standard SR is 5/m^2^. For Yangzhou geese, from 4 to 10 weeks, the standard SR is 5/m^2^.

## Conclusion

This review identified a wide range of documented examples of the effect of stocking rate on the welfare indices and productive traits of ducks and geese. According to the available information, it can be concluded that:

- Using an optimal stocking rate can improve behaviors, productivity (meat-egg), and meat quality for waterfowl.
- Fish-waterfowl production system could be a promising and sustainable solution for increasing waterfowl production, maintaining birds’ welfare, saving energy, and reducing the water footprint of waterfowl meat. It is worth noting that the best SR is 0.5 goose/m^2^ for the adults.
- Considering previous studies, we suggested SR standard for some duck and goose breeds is to maintain the best production-welfare balance.

## Data Availability

Not applicable.
